# The relationship between eHealth literacy, social media self-efficacy and health communication intention among Chinese nursing undergraduates: A cross-sectional study

**DOI:** 10.3389/fpubh.2022.1030887

**Published:** 2022-10-31

**Authors:** Huiping Sun, Lin Qian, Mengxin Xue, Ting Zhou, Jiling Qu, Jingxin Zhou, Junchao Qu, Siqi Ji, Yuan Bu, Yicheng Hu, Shaung Wu, Yuhui Chen, Jiachun You, Yongbing Liu

**Affiliations:** School of Nursing, School of Public Health, Yangzhou University, Yangzhou, China

**Keywords:** nursing undergraduates, eHealth literacy, social media self-efficacy, health communication, health communication intention

## Abstract

**Background:**

With the popularization of the Internet, it has become possible to widely disseminate health information *via* social media. Medical staff's health communication through social media can improve the public's health literacy, and improving the intention of health communication among nursing undergraduates is of great significance for them to actively carry out health communication after entering clinical practice.

**Objective:**

To explore the relationship among eHealth literacy, social media self-efficacy, and health communication intention and to determine the mediating role of social media self-efficacy in the relationship between eHealth literacy and health communication intention.

**Design:**

A cross-sectional descriptive correlation design was used in this study.

**Participants:**

Stratified cluster sampling was used to select 958 nursing students from four nursing colleges in Jiangsu Province, China, from June to July 2021.

**Methods:**

Data were collected using the eHealth Literacy Scale, the Social Media Self-efficacy Scale, and the Health Communication Intention Questionnaire. Sociodemographic data were also collected. Correlation analysis and regression analysis were used to determine the relationship between eHealth literacy, social media self-efficacy, and health communication intention.

**Results:**

Health communication intention is positively correlated with eHealth literacy and social media self-efficacy. There is a significant positive correlation between eHealth literacy and health communication intention (β = 0.57, *p* < 0.001), and social media self-efficacy played a mediating role in the influence of eHealth literacy on health communication intention (the mediating effect accounted for 37.2% of the total effect).

**Conclusion:**

The study found that eHealth literacy and social media self-efficacy had an impact on health communication intention. Because there is a correlation between eHealth literacy and social media self-efficacy and health communication intention, in order to promote health communication intention of nursing students, it is also important to cultivate eHealth literacy and social media self-efficacy of nursing students. In view of these results, targeted educational programs must be developed to improve eHealth literacy and social media self-efficacy among nursing undergraduates, thereby promoting their health information transmission.

## Introduction

Health communication is a kind of behavior that transforms the results of medical research into public health knowledge and aims to reduce the morbidity and mortality due to diseases and effectively improve the quality of life and health of a community or country through attitude and behavior change (1). With the improvement of public health awareness and the advent of the Internet era, the Internet has become the main source of public health information (2). As of June 2019, China had 854 million Internet users and an Internet penetration rate of 61.2% (3). With the rapid development of new media, health information can be transmitted through different media (4). Social media enables people to spread health information by creating and sharing content *via* a wide variety of applications (such as WeChat, Weibo, and blogs) (5). Studies have found that doctor-patient interactions on social media can have a significant impact on patients' health behaviors through knowledge, self-efficacy, and outcome expectations (6, 7). Among the active users of new media who pay attention to health information, 92% will change their health behavior after reading health information, wherein 71% of users act immediately, and 45% are influenced by the message (8).

However, the quality of health information obtained through social media varies greatly, and health professionals need to engage in health communication *via* relevant media to improve the reliability of health information (9, 10). Studies have shown that people are more likely to trust health information transmitted by professionals, such as medical staff (11). At present, it is widely believed that effective communication plays an important role in health care in medical and academic circles. Health communication is not an insignificant addition to the medical process but rather lies at the core of the medical process (12). By disseminating health knowledge through social media, medical staff can improve people's health literacy and, thus, improve their work efficiency (13). Nurses play an important role in the dissemination of health information (14). In order to promote the use of social media by nurses to share expertise and acquire knowledge in their field of work in their free time, the Finnish Nurses Association has developed guidelines for social media use (15). Therefore, the intention of health communication in this study is the intention of using social media to spread health knowledge. However, the study found that nurses are mostly invisible in the media due to a lack of media literacy and communication intention (16, 17). Therefore, there is an urgent need to improve nurses' intention to communicate about health on social media to change this situation. Some studies have pointed out that age will affect the willingness to learn to use social media, the older the age (18), the lower the willingness to learn to use social media, and the greater the work pressure of nurses, it is relatively difficult to find free time to learn how to use social media to spread health knowledge (19). Nursing students are the main force of the nursing industry in the future. They have a good medical knowledge base and also need to assume the responsibility of spreading health information (20). At the same time, cultivating the ability of using social media to spread health information is conducive to better spreading health knowledge after entering clinical practice.

The purpose of health communication activities is to improve people's health levels. To ensure the scientific nature of the health information, disseminators need to have good health literacy. In the information age, eHealth literacy is an important part of health literacy. Some studies have pointed out that the level of eHealth literacy will affect the intention of users to spread health information (21). eHealth literacy is the ability to seek, discover, understand, and evaluate health information in electronic media and to share access to this information to solve health problems (22). There are six core types of literacy: traditional literacy (basic reading and writing skills are essential to derive meaning from text-filled resources), health literacy (patients with sufficient health literacy can read, understand, and act on healthcare information), information literacy (an information literate person knows which potential resources to consult for information on a particular topic, can develop an appropriate search strategy, and can filter the results to extract relevant knowledge), scientific literacy (Does scientific literacy place health research findings in proper context, informing consumers about how science is done and the largely incremental process of discovery, as well as the limitations and opportunities that research may present), media literacy (enabling people to place messages in a social and political context and to consider issues such as how markets, audience relations, and the media form itself shape the message conveyed), and computer literacy (including the ability to adapt to new technologies and software, including absolute and relative access to e-health resources). Making the most of eHealth resources requires both analytical (traditional literacy, media literacy, and information) and context-specific skills (computer literacy, scientific literacy, and health literacy) (23). Previous literature suggests that users' intentions to share health information on social media may be related to their level of eHealth literacy (21, 24, 25). The Integrative Model of E-Health Use (ImeHU) (26) also indicates that eHealth literacy contributes to health information effectiveness and eHealth practices (i.e., eHealth use behaviors, such as searching for health knowledge on the Internet and using social networks to communicate health knowledge with others). Health communication *via* social media is a key step in promoting healthy behavior change. However, empirical studies on eHealth literacy and its impact on health communication intentions are still lacking, so we propose the following hypotheses:

Hypothesis 1: There is a positive relationship between eHealth literacy and the intention of nursing undergraduates to participate in health communication.

The social cognitive model proposed by Bandura (27) points out that self-efficacy can significantly affect the effort level of individuals to take risks. Self-efficacy is a key structure in social cognitive theory, describing a person's confidence in their ability to complete an action or a behavior that will lead to the desired outcome. Technology-related self-efficacy can affect job performance. Social media self-efficacy is a person's perceived ability to achieve their desired results in a social media environment (18). A number of studies have shown that self-efficacy can positively influence health transmission behavior (28–30). However, there are few studies on social media self-efficacy and eHealth literacy. This study aims to explore whether eHealth literacy can influence the intention of health communication among nursing undergraduates through social media self-efficacy. Therefore, we propose the following hypothesis:

Hypothesis 2: The higher the level of social media self-efficacy of nursing undergraduates, the stronger their intention to communicate about health.

**Figure 1:**
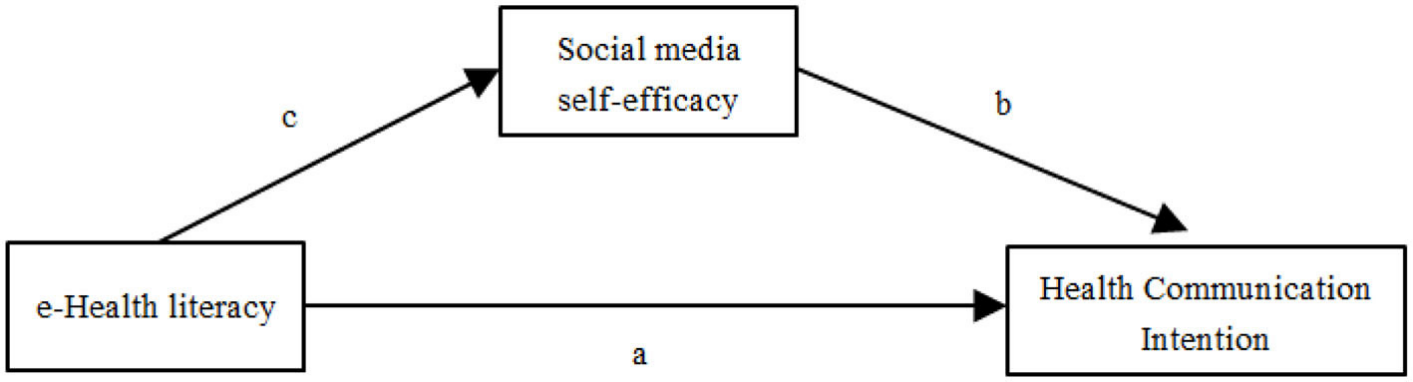
Hypothesized model. a: There is a positive relationship between eHealth literacy and the intention of nursing undergraduates to participate in health communication (hypothesis 1); b: The higher the level of social media self-efficacy of nursing undergraduates, the stronger their intention to communicate about health (hypothesis 2); c: Social media self-efficacy mediates the impact of eHealth literacy on health communication intention (hypothesis 3).

Hypothesis 3: Social media self-efficacy mediates the impact of eHealth literacy on health communication intention. The hypothesized model of this study is shown in Figure 1.

## Methods

### Study design

This is a cross-sectional descriptive study examining the effects of eHealth literacy and social media self-efficacy on health communication intentions among nursing undergraduates.

### Participants

Participants were recruited through stratifiedcluster sampling from students at four nursing schools in a province in China, and data were collected between June 2021 and July 2021. The inclusion criteria included: (1) voluntary participation in the study; (2) being a full-time undergraduate student majoring in nursing. Through the power analysis performed to determine the sample size, the sample size was calculated as *n* = 188, with a 0.30 effect size, 99% power, and 0.05 margin of error. The sample size of this study was 958.

### Data collection and ethical considerations

This study was approved by the Ethics Committee of the School of Nursing at Yangzhou University (No: YZUHL2021026). We sent questionnaires to teachers at four schools and commissioned them to distribute the questionnaires to nursing undergraduates. Participants completed a survey *via* the online survey platform Wenjuangxing (www.wjx.cn), a website that allows you to create electronic questionnaires for free. Online surveys help ensure that the responses submitted do not contain missing data. One device, one account, and one IP address can complete only one questionnaire. Completing the online questionnaire was considered a voluntary agreement to participate in the study.

### Instruments

The questionnaire collected the demographic information of nursing students (gender, year in nursing school, monthly household income, understanding of health communication, media-related training, nursing experience, and intention to change major), as well as their eHealth literacy, social media self-efficacy, and health communication intention.

The eHealth Literacy Scale was used to evaluate the eHealth literacy of nursing students. The scale was originally developed by Norman (31) and adapted by Tang et al. (32) due to cultural differences and the development of Web 2.0 technology. The revised scale consists of 20 questions, including three dimensions: health information acquisition ability (items 1–7), health information evaluation ability (items 8–15), and health information practice ability (items 16–20). The Cronbach's α in this study was 0.96, showing good homogeneity and internal consistency. A 5-point Likert scale was used for each project (from 1 = strongly disagree to 5 = strongly agree), and the total score was between 20 and 100. The higher the score, the higher the students' eHealth literacy level.

The Social Media Self-efficacy Scale was adapted by Qiaodan et al. (33), based on the Social Media Self-efficacy Scale developed by Alber et al. (34), with a total of 24 questions aimed at evaluating nursing undergraduates' confidence in their social media ability. The Cronbach's α in this study was 0.98. A 6-point Likert scale (from 1 = very low confidence to 6 = very high confidence) was used, and the total score ranged from 24 to 144. The higher the score was, the better the nursing undergraduates' social media self-efficacy was.

According to the purpose of this study, the Science Communication Intention Questionnaire (35), compiled by Wu et al., was adapted into the Health Communication Intention Questionnaire, consisting of three items. A 5-point Likert scoring item was used to evaluate the degree to which nursing undergraduates were willing to engage in health communication. The Cronbach's α in this study was 0.89.

### Statistical analysis

Descriptive statistics were used to analyze general information about participants, such as means, ranges, standard deviations, and percentages. A *t*-test and an ANOVA were used to analyze differences in the health communication intentions of participants, and a Pearson correlation was used to analyze the correlation among eHealth literacy, health communication self-efficacy, and health communication intention. The Process V3 plug-in, developed by Andrew F. Hayes, was used to analyze the mediating effect, and *P* < 0.05 was considered statistically significant. The bias-corrected Bootstrap method was used to test the mediation effect, and the 95% confidence interval (CI) of the mediation effect was estimated. If the 95% CI does not contain 0, it indicates that the mediation effect is significant. SPSS 26.0 software was used to sort and analyze the data.

## Results

### Participants' characteristics

Among the participants, 235 (24.5%) were male; 292 students (30.5%) were in their 1st year, 295 students (30.8%) were in their 2nd year, 238 students (24.8%) were in their 3rd year, 133 students (13.9%) were in their 4th year; 626 people (65.3%) had a monthly household income of more than 5,000 yuan (783 dollars); only 81 students (8.5%) had received media-related training; 305 (31.8%) had nursing experience (Table 1). As shown in Table 2, the average scores of eHealth literacy, social media self-efficacy, and health communication intention of nursing undergraduates were 74.01 ± 13.36, 92.74 ± 20.82, and 11.14 ± 1.93, respectively.

**Table 1:** Differences in general characteristics of participants' intention to transmit health information (*N* = 958).

**Characteristic**	**Category**	***N* (%)**	**Mean ±SD**	***t*/*F***	***P***
Gender	Male	235 (24.5)	11.01 ± 0.15	−1.04	0.301
	Female	723 (75.5)	11.18 ± 0.07		
Academic year	First	292 (30.5)	10.90 ± 0.12	3.23	0.022
	Second	295 (30.8)	11.20 ± 0.11		
	Third	238 (24.8)	11.14 ± 0.11		
	Fourth	133 (13.9)	11.50 ± 0.15		
Monthly household income	≤ 5,000	332 (24.7)	10.95 ± 0.10	−2.23	0.026
	>5,000	626 (65.3)	11.23 ± 0.08		
Knowledge of health communication	Completely unknown	32 (3.3)	10.59 ± 0.59	19.91	< 0.001
	Not very familiar	129 (13.5)	10.71 ± 0.16		
	Average	400 (41.8)	10.78 ± 0.09		
	Known	323 (33.7)	11.47 ± 0.10		
	Very well-known	74 (7.7)	12.58 ± 0.24		
Media related training	Yes	81 (8.5)	11.96 ± 0.21	4.07	< 0.001
	No	877 (91.5)	11.06 ± 0.06		
Nursing experience	Yes	305 (31.8)	11.48 ± 0.10	3.93	< 0.001
	No	653 (68.2)	10.97 ± 0.08		
Intention to change major	Yes	538 (56.2)	11.08 ± 0.08	−1.05	0.296
	No	420 (43.8)	11.20 ± 0.09		

SD, standard deviation.

**Table 2:** Participants' scores for the eHEALS, social media self-efficacy, and health communication intention (*N* = 958).

**Measure**	**Mean**	**SD**	**Minimum**	**Maximum**
eHEALS	74.01	13.36	20.00	100.00
Social media Self-efficacy	92.74	20.82	24.00	144.00
Health Communication Intention	11.14	1.93	3.00	15.00

eHEALS, the eHealth Literacy Scale; SD, standard deviation.

### Differences in each participant's health communication intention

Health communication intention scores by nursing school year (*F* = 3.23, *P* = 0.022), monthly family income (*t* = −2.23, *P* = 0.026), knowledge of health communication (*F* = 19.91, *p* < 0.001), media-related training (*t* = 4.07, *p* < 0.001), whether the student had any internship experience (*t* = 3.93, *p* < 0.001), and the results are shown in Table 1. Among them, the intention of engaging in health communication among year 1 students was higher than that of students in other years. Students with a high monthly household income have strong intentions to engage in health communication. The higher their understanding of health communication, the stronger their intention to engage in health communication. The students with media-related training had higher intentions of engaging in health communication than those without. In addition, students with internship experience were more willing to spread health information than those without internship experience.

### Correlations among variables

Table 3 shows the binary correlations between eHealth literacy, social media self-efficacy, and health communication intention. Health communication intention was positively correlated with both eHealth literacy (*r* = 0.59, *p* < 0.001) and social media self-efficacy (*r* = 0.56, *p* < 0.001); eHealth literacy was also positively correlated with social media self-efficacy (*r* = 0.68, *p* < 0.001).

**Table 3:** Bivariate correlations among measures (*N* = 958).

**Measure**	**eHEALS**	**Social media self-efficacy**	**Health communication intention**
eHEALS	1		
Social Media Self-efficacy	0.68**	1	
Health Communication Intention	0.59**	0.56**	1

eHEALS, the eHealth Literacy Scale.

^**^p ≤ 0.001.

### Mediating role of social media self-efficacy in the relationship between eHealth literacy and health communication intention

**Figure 2:**
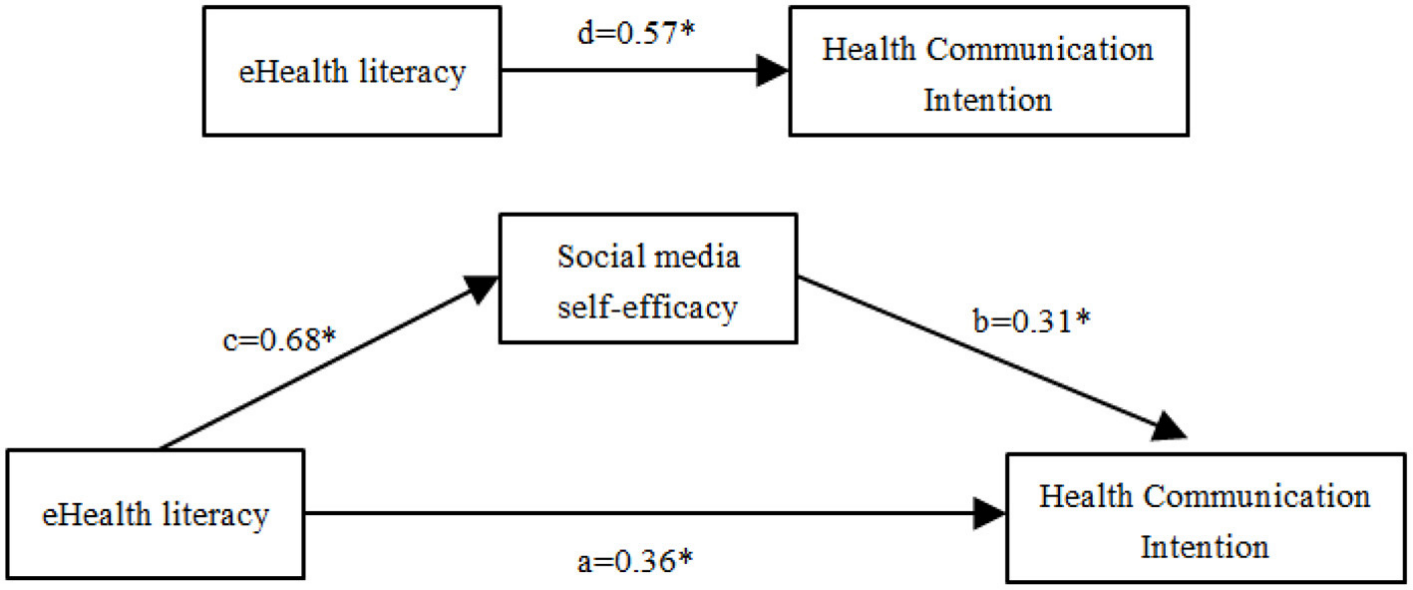
Mediation model shows the direct effect and path coefficients that link eHealth literacy and health communication intention *via* social media self-efficacy (*n* = 958). a: the unstandardized regression coefficient between eHealth literacy on health communication intention. b: the unstandardized regression coefficient between social media self-efficacy and health communication intention; c: the unstandardized regression coefficient of eHealth literacy on social media self-efficacy; d: the total effect between eHealth literacy and health communication intention. *:*p* ≤ 0.001.

In the first step, there was a significant positive correlation between eHealth literacy and social media self-efficacy (β = 0.68, *p* < 0.001; Table 4), and the explanatory power was 48.2%. In the second step, there is a significant positive correlation between eHealth literacy and health communication intention (β = 0.57, *p* < 0.001), and the explanatory power was 35.1%. In the third step, social media self-efficacy has a significant positive correlation with health communication intention (β = 0.31, *p* < 0.001), while eHealth literacy has a significant positive correlation with social media self-efficacy (β = 0.36, *p* < 0.001), and the explanatory power was 40.1% (Table 4 and Figure 2). The Bootstrap method was used to further verify the mediating effect of social media self-efficacy. The 95% CI of the direct effect and indirect effect of eHealth literacy on health communication intention did not contain zero. In general, the mediating effect model of social media self-efficacy was established, and the mediating effect value was 0.21, accounting for 37.2% of the total variation. The results are shown in Table 5.

**Table 4:** Mediating role of social media self-efficacy in the relationship between eHealth literacy and health communication intention (*N* = 958).

**Variable**	**Step 1. Social media self-efficacy**				**Step 2. Health communication intention**				**Step 3. Health communication intention**			
	***B***	***β***	***t***	***p***	***B***	***β***	***t***	***P***	***B***	***β***	***t***	***p***
Academic year	−0.54	−0.03	−0.94	0.348	0.06	0.03	1.09	0.276	0.08	0.04	1.40	0.161
Monthly household income	3.84	0.18	3.74	0.000	0.10	0.05	0.95	0.341	−0.01	0.00	−0.09	0.930
Knowledge of health communication	−0.21	−0.01	−0.36	0.719	0.02	0.01	0.28	0.780	0.02	0.01	0.39	0.694
Media related training	−4.29	−0.21	−2.42	0.016	−0.39	−0.20	−2.10	0.036	−0.26	−0.14	−1.48	0.140
Nursing experience	2.92	0.14	2.30	0.022	−0.05	−0.03	−0.40	0.691	−0.14	−0.07	−1.08	0.283
eHealth literacy	1.07	0.68	26.55	< 0.001	0.08	0.57	19.80	< 0.001	0.05	0.36	9.80	< 0.001
Social media self-efficacy									0.03	0.31	8.89	< 0.001
*R* ^2^	0.48				0.35				0.40			
*F*	147.82				85.76				90.84			
*P*	< 0.001				< 0.001				< 0.001			

B, The standardized regression coefficient; β, The unstandardized regression coefficient.

**Table 5:** Mediation effect Bootstrap results.

**Effect of type**	**Effect**	**SE**	**LLCI**	**ULCI**	**Relative effect value**
Total effect	0.57	0.04	0.49	0.65	
Direct effect	0.36	0.05	0.26	0.46	62.80%
Indirect effect	0.21	0.03	0.16	0.26	37.20%

SE, Standard Error; CI, Confidence Interval; LLCI, Lower Limit Confidence Interval; ULCL, Upper Limit Confidence Limit Interval.

## Discussion

This study investigated the present situation of nursing undergraduates' eHealth literacy, social media self-efficacy, and their intention to engage in health communication, and probed into the relationship among the eHealth literacy, social media self-efficacy, and health communication intention, investigating the mediating effect of social media self-efficacy on eHealth literacy and health communication intention. Research results show that nursing students' willingness to communicate health is at a medium level, which may be related to the majority of nursing students in China who want to leave the nursing profession (36–38), although our research results show no significant impact on health communication willingness. In this study, nursing undergraduates were more willing to participate in health communication than the scientists investigated by Wen-xi and Ting (35). The reason for this phenomenon may be determined by the characteristics of the profession. Nurses often have two-way communication with patients in their daily work to cultivate patients' behavior of sharing health knowledge.

Our results show that eHealth literacy has a significant positive correlation with the intention to engage in health communication, indicating that nursing undergraduates with higher eHealth literacy have a stronger intention to engage in health communication. Hypothesis 1 is, therefore, verified. Zhao et al. (39) found that users with high levels of eHealth literacy were more willing to share health articles on social media, which is consistent with our findings. As pointed out in the previous article, eHealth literacy consists of multiple dimensions, such as the basic knowledge and information search ability of nursing students from health literacy and information search ability. Some studies have pointed out that people with high health literacy are more likely to share health information (40), and 40% of people who have searched for health information will share health information (41). Nursing undergraduates with high e-health literacy may have good health literacy to understand the health information needs of patients, and may also have good information literacy to effectively use information technology for information retrieval. All of these factors enhance students' perceptual behavior control and further promote their communication intentions. In our study, the eHealth literacy score of nursing undergraduates was higher than that of nursing undergraduates in Shandong Province, China, surveyed by Guo and Xin (42) and the students' scores in Tang's adapted tool (32). One possible reason is that through the study of disease prevention, health care, and other theories, the professional knowledge reserve of undergraduate nursing students can help them identify and evaluate eHealth resources (43). However, considering that medical students may have inflated self-efficacy and systematic evaluation, they may lack the actual ability to locate and evaluate high-quality health information on the Internet (44). Therefore, medical education needs to set up corresponding courses to cultivate students' ability to search evidence-based health information on the Internet, enabling them to embed eHealth information into their behavior and lifestyle.

The study found that social media self-efficacy also has a significant positive correlation with health communication intention, which verifies Hypothesis 2 and is consistent with previous research results on self-efficacy and behavioral intention (41). Ajjan found in his research on entrepreneurial intentions that social media self-efficacy could positively predict perceived behavioral control, thus promoting entrepreneurial intentions (45). This also confirms the important role of social media self-efficacy in promoting health communication intentions. The low social media self-efficacy scores of nursing undergraduates in this study indicate that they lack sufficient skills to search for reliable information on the Internet or social media, which may be related to the fact that nursing colleges in China offer relevant information skills courses but do not pay enough attention to them. The low self-efficacy regarding social media use among nursing undergraduates may be due to the lack of adequate social media channels and limited opportunities to use social media to communicate health knowledge (18). Some studies have also found that experience in producing social media content contributes to social media self-efficacy, helps individuals build confidence in their ability to successfully find the specific information they need online, and enables them to perceive their social media skills accurately (9, 18). Such experience can be obtained through practice, training, and guidance. It is suggested that nursing colleges should pay attention to the training of students' information skills, carry out corresponding health communication activities, and provide a platform for students to spread health knowledge effectively.

Another important finding of this study is that social media self-efficacy plays a mediating role between eHealth literacy and health communication intention, accounting for 37.2% of the total effect. Hypothesis 3 is also verified. The results suggest that institutions can prioritize the social media self-efficacy of nursing students to ultimately improve their willingness to communicate health. A high eHealth literacy among nursing undergraduate students in the process of retrieving health information is a good way to observe others' successful health communication on social media. On the one hand, this is due to the effect of alternative experiences and varying media self-efficacy levels; on the other hand, it can also stimulate positive emotions among nursing undergraduates so as to enhance their self-efficacy level, eventually promoting health communication (30). This suggests that nursing colleges can show students positive cases of health communication from classmates or teachers and set up corresponding skill training courses to enhance their confidence in health communication and encourage them to actively engage in it, which is of great significance to improving the intention to engage in health communication among nursing undergraduates.

There are some limitations to this study. This study is only a cross-sectional study, so causal relationships cannot be inferred from the correlations between variables. Therefore, a longitudinal design should be considered in future studies. Only investigated nursing undergraduates from four universities in Jiangsu Province, China, and its results may not be applicable to nursing undergraduates from other regions, so its stability among intercultural nursing undergraduates needs to be explored in the future. All the participants participated voluntarily, and those nursing students with higher awareness of eHealth literacy may be more willing to participate in our study, and students' intentions to engage in health communication were self-reported, which may have been affected by social expectations, which may cause selection bias. In the future, actual health communication behaviors should be considered for research. Finally most of the participants are female students, and nearly half of them have the idea of switching majors. However, this reflects the gender ratio and brain drain of nursing students in our country.

## Conclusions

In China, health communication plays a vital role in improving public health literacy. This study supports the mediating role of social media self-efficacy between eHealth literacy and health communication intention. The study found that eHealth literacy and social media self-efficacy had an impact on health communication intention. Because there is a correlation between eHealth literacy and social media self-efficacy and health communication intention, in order to promote health communication intention of nursing students, it is also important to cultivate eHealth literacy and social media self-efficacy of nursing students. In view of these results, targeted educational programs must be developed to improve eHealth literacy and social media self-efficacy among nursing undergraduates, thereby promoting their health information transmission.

## Data availability statement

The raw data supporting the conclusions of this article will be made available by the authors, without undue reservation.

## Ethics statement

The studies involving human participants were reviewed and approved by the School of Nursing at Yangzhou University (No: YZUHL2021026). The patients/participants provided their written informed consent to participate in this study.

## Author contributions

HS and LQ: writing and preliminary manuscript draft preparation and research. MX: software and data management. JiQ and TZ: investigation and verification. JZ and JuQ: supervision and verification. SJ, YB, YH, SW, YC, and JY: investigation. YL: conceptualization, methodology, proofreading, and editing. All authors contributed to the article and approved the submitted version.

## Funding

This study was supported by Innovation and Entrepreneurship Training Program for College Students of Jiangsu Province (Nos. 202111117020Z and 202211117010Z) and the 2019 Jiangsu Province Geriatric Teaching and Learning Resource Library Sub-Library Project.

## Conflict of interest

The authors declare that the research was conducted in the absence of any commercial or financial relationships that could be construed as a potential conflict of interest.

## Publisher's note

All claims expressed in this article are solely those of the authors and do not necessarily represent those of their affiliated organizations, or those of the publisher, the editors and the reviewers. Any product that may be evaluated in this article, or claim that may be made by its manufacturer, is not guaranteed or endorsed by the publisher.

## References

[1] Rogers EM. The field of health communication today: an up-to-date report. J Health Commun. (1996) 1:15–23. 10.1080/108107396128202 10947350

[2] Tariq A Khan SR Basharat A. Internet use, eHealth literacy, and dietary supplement use among young adults in Pakistan: cross-sectional study. J Med Internet Res. (2020) 22:e17014. 10.2196/17014 32519974 PMC7315369

[3] Yu Z. CNNIC releases the 44th statistical report on internet development in China. Civil-Military Integr Cyberspace. (2019) 2019:30–1.

[4] Zhang D Shi Z Hu H Han GK. Classification of the use of online health information channels and variation in motivations for channel selection: cross-sectional survey. J Med Internet Res. (2021) 23:e24945. 10.2196/24945 33687342 PMC7988389

[5] Chen J Wang Y. Social media use for health purposes: systematic review. J Med Internet Res. (2021) 23:e17917. 10.2196/17917 33978589 PMC8156131

[6] Wu T Deng Z Feng Z Gaskin DJ Zhang D Wang R. The effect of doctor-consumer interaction on social media on consumers' health behaviors: cross-sectional study. J Med Internet Res. (2018) 20:e73. 10.2196/jmir.9003 29490892 PMC5852273

[7] Chen Z Hunag H Chen J. An empirical study on the influence of health communication information on audience's health behavior: an experiment based on dietary behavior tendency. Modern Commun. (2016) 38:52–7. 10.3969/j.issn.1007-8770.2016.07.010

[8] Jin X Zhang D Fen H. An empirical study on health information dissemination effect in mobile social media. Informat Sci. (2018) 36:129–35. 10.13833/j.issn.1007-7634.2018.09.023

[9] Hocevar KP Flanagin AJ Metzger MJ. Social media self-efficacy and information evaluation online. Comput Human Behav. (2014) 39:254–62. 10.1016/j.chb.2014.07.020

[10] Hopf H Krief A Mehta G Matlin SA. Fake science and the knowledge crisis: ignorance can be fatal. R Soc Open Sci. (2019) 6:190161. 10.1098/rsos.190161 31218057 PMC6549953

[11] Eysenbach G Credibility Credibility of health information and digital media: New perspectives and implications for youth. MacArthur Foundation Digital Media and Learning Initiative (2008).

[12] Liu H. On the positive communication potential of we media and doctor-patient relationship. Modern Commun. (2018) 40:44–8.32417891

[13] Dailah HG Naeem M. A social media organizational productivity model: insights from public health professionals. J Med Internet Res. (2021) 23:e23792. 10.2196/23792 33949965 PMC8135021

[14] Henly SJ. Health communication research for nursing science and practice. Nurs Res. (2016) 65:257–8. 10.1097/NNR.0000000000000171 27362511

[15] Arifulla D Olli J Merasto M. Nurses guidelines for using social media by Finnish Nurses Association. Stud Health Technol Inform. (2016) 225:617–8. 10.3233/978-1-61499-658-3-617 27332278

[16] Chaffee M. Health communications: nursing education for increased visibility and effectiveness. J Prof Nurs. (2000) 16:31–8. 10.1016/S8755-7223(00)80009-5 10659517

[17] Ye H Zhang C Wu y Yang H. Investigation on the status quo of WeChat health popularization and education by nurses in some hospitals in Zhejiang Province. Med Soc. (2018) 31:24–7. 10.13723/j.yxysh.2018.05.009

[18] Alber JM Paige S Stellefson M Bernhardt JM. Social media self-efficacy of health education specialists: training and organizational development implications. Health Promot Practice. (2016) 17:915–21. 10.1177/1524839916652389 27234984

[19] Kazemi SS Tavafian SS Hidarnia A Montazeri A. Exploring nurses' experiences of social media and in-person educational interventions for professional development: a qualitative study. BMC Nurs. (2022) 21:126. 10.1186/s12912-022-00903-4 35610638 PMC9128214

[20] Tang Q Song GX. Some thoughts on the socialization trend of health science popularization. Chin J Medical Sci Res. (2005) 2005:252–4. 10.3760/cma.j.issn.1006-1924.2005.04.024

[21] Chi Y. What sources to rely on: Laypeople's source selection in online health information seeking. In: Proceedings of the 2018 Conference on Human Information Interaction & Retrieval. New Brunswick, NJ: Association for Computing Machinery (2018). p. 233–6. 10.1145/3176349.3176881

[22] Oh H Rizo C Enkin M Jadad A. What is eHealth (3): a systematic review of published definitions. J Med Internet Res. (2005) 7:e1. 10.2196/jmir.7.1.e1 15829471 PMC1550636

[23] Norman CD Skinner HA. eHealth literacy: essential skills for consumer health in a networked world. J Med Internet Res. (2006) 8:e9. 10.2196/jmir.8.2.e9 16867972 PMC1550701

[24] Zhang X Yan X Cao X Sun Y Chen H She J. The role of perceived e-health literacy in users' continuance intention to use mobile healthcare applications: an exploratory empirical study in China. Informat Technol Dev. (2018) 24:198–223. 10.1080/02681102.2017.1283286

[25] Li X Liu Q. Social media use, eHealth literacy, disease knowledge, and preventive behaviors in the COVID-19 pandemic: cross-sectional study on Chinese Netizens. J Med Internet Res. (2020) 22:e19684. 10.2196/19684 33006940 PMC7581310

[26] Bodie GD Dutta MJ. Understanding health literacy for strategic health marketing: eHealth literacy, health disparities, and the digital divide. Health Mark Q. (2008) 25:175–203. 10.1080/07359680802126301 18935884

[27] Bandura A Freeman WH Lightsey R. Self-efficacy: the exercise of control. J Cogn Psychother. (1999) 1999:158–66. 10.1891/0889-8391.13.2.158

[28] Huang H Liu J Chen Y Yang X. The effect of nurses' general self-efficacy and organizational trust on knowledge sharing. J Nurses Training. (2021) 36:1034–7. 10.16821/j.cnki.hsjx.2021.11.015

[29] Kim H Lee J Oh SE. Individual characteristics influencing the sharing of knowledge on social networking services: online identity, self-efficacy, and knowledge sharing intentions. Behav Inf Technol. (2020) 39:379–90. 10.1080/0144929X.2019.1598494

[30] Alshahrani H Rasmussen Pennington D. “Why not use it more?” Sources of self-efficacy in researchers' use of social media for knowledge sharing. J Document. (2018) 74:1274–92. 10.1108/JD-04-2018-0051

[31] Norman CD Skinner HA. eHEALS: the eHealth literacy scale. J Med Internet Res. (2006) 8:e27. 10.2196/jmir.8.4.e27 17213046 PMC1794004

[32] Tang Z Wang F Fu H. Development and evaluation of electronic media health literacy scale for college students. Chin J Health Educ. (2014) 30:35–8. 10.16168/j.cnki.issn.1002-9982.2014.01.002

[33] Qiaodan L Qinghong S Qingbo L Lin X Jing L. Chinese version of Social Media Competency Inventory and its reliability and validity test. Chin Nurs Res. (2021) 35:1892–8. 10.12102/j.issn.1009-6493.2021.11.003

[34] Alber JM Bernhardt JM Stellefson M Weiler RM Anderson-Lewis C Miller MD. Designing and testing an inventory for measuring social media competency of certified health education specialists. J Medical Internet Res. (2015) 17:e221. 10.2196/jmir.4943 26399428 PMC4642407

[35] Wen-xi W Ting Z. Investigating factors affecting scientists' willingness to engage in online science communication: an empirical survey study. J Northeast Normal Univ. (2021) 2021:111–6. 10.16164/j.cnki.22-1062/c.2021.02.015

[36] Gong Z Li W Bu H He M Hou H Ma T. Impact of COVID-19 pandemic on the professional intention of medical and related students. BMC Med Educ. (2021) 21:484. 10.1186/s12909-021-02922-2 34503514 PMC8428501

[37] Tang M Sun Y Zhang K Luo R Liu R Liu Y. Associated factors of professional identity among nursing undergraduates during COVID-19: a cross-sectional study. Int J Nurs Sci. (2022) 9:107–13. 10.1016/j.ijnss.2021.09.005 34567827 PMC8452454

[38] Bai W Xi HT Zhu Q Wang Z Han L Chen P. Changes in nursing students' career choices following the COVID-19 pandemic in China. Front Psychiatry. (2021) 12:657021. 10.3389/fpsyt.2021.657021 33927657 PMC8076572

[39] Zhao HP Fu SX Chen XY. Promoting users' intention to share online health articles on social media: the role of confirmation bias. Informa Process Manag. (2020) 57:102354. 10.1016/j.ipm.2020.102354 32834400 PMC7368841

[40] Crook B Stephens KK Pastorek AE Mackert M Donovan EE. Sharing health information and influencing behavioral intentions: the role of health literacy, information overload, and the internet in the diffusion of healthy heart information. Health Commun. (2016) 31:60–71. 10.1080/10410236.2014.936336 25668744

[41] Niu Z Willoughby J Zhou R. Associations of health literacy, social media use, and self-efficacy with health information-seeking intentions among social media users in China: cross-sectional survey. J Med Internet Res. (2021) 23:e19134. 10.2196/19134 33629955 PMC7952238

[42] Guo M Xin H. Correlation analysis of electronic health literacy and health self-management ability in 578 undergraduate nursing students. J Nurs. (2019) 26:65–8. 10.16460/j.issn1008-9969.2019.23.065

[43] Turan N Özdemir NG Çulha Y Aydin GO Kaya H Aşti T. The effect of undergraduate nursing students' e-Health literacy on healthy lifestyle behavior. Glob Health Promot. (2021) 28:6–13. 10.1177/1757975920960442 33023383

[44] Stellefson M Hanik B Chaney B Chaney D Tennant B Chavarria EA. eHealth literacy among college students: a systematic review with implications for eHealth education. J Medical Internet Res. (2011) 13:e102. 10.2196/jmir.1703 22155629 PMC3278088

[45] Ajjan H Fabian F Tomczyk D Hattab H. Social media use to support entrepreneurship in the face of disruption. J Dev Entrepreneur. (2015) 20:15500144. 10.1142/S1084946715500144

